# The discovery of the genus *Spasskia* Belokobylskij, 1989 (Hymenoptera: Braconidae) in China, with description of a new species

**DOI:** 10.1093/jis/14.1.119

**Published:** 2014-09-01

**Authors:** Cheng-jin Yan, Jun-hua He, Xue-xin Chen

**Affiliations:** State Key Laboratory of Rice Biology and Ministry of Agriculture Key Lab of Agricultural Entomology, Institute of Insect Sciences, Zhejiang University, Hangzhou 310058, China

**Keywords:** Braconidae, Helconinae, Hymenoptera, key, new record

## Abstract

The genus *Spasskia*Belokobylskij, 1989 (Hymenoptera: Braconidae: Helconinae) is reported for the first time from China. Two species, namely *Spasskia brevicarinata* Yan et Chen sp. n.and *Spasskia indica* Singh, Belokobylskij et Chauhan, 2005 are described and illustrated. A key to the species of this genus is updated to include the new species.

## Introduction

Three species of the genus *Spasskia*Belokobylskij, 1989 (Hymenoptera: Braconidae: Helconinae) have been described previously: *S. anastasiae* Belokobylskij, *S. indica* Singh, Belokobylskij et Chauhan, and *S. sigalphoides* Belokobylskij (type species) ( Belokobylskij 1989 , 1998; Belokobylskij and Ku 1998 ; Singh et al. 2005 ). *S. indica* is a parasitoid of *Chlorophorus strobilicola* Champion (Coleoptera: Cerambycidae) infesting second- and third-year cones of *Pinus roxburghii* Sargent (Pinales: Pinaceae) ( Singh et al. 2005 ).

During a study of Chinese braconids, a new *Spasskia* species and a previously described *Spasskia* species were discovered. These are the first records of *Spasskia* in China. The new species, *S. brevicarinata* sp. n., is described and illustrated, and the newly recorded species, *S. indica,* is illustrated. An updated key to the genus *Spasskia* is also presented.

## Materials and Methods

The terminology and measurements used follow van Achterberg (1988 , 1993). Additional sources for the description of sculpture and setation are Belokobylskij (1998) and Singh et al. (2005) . The following abbreviations are used: POL, postocellar line; OOL, ocular-ocellar line; OD, maximum diameter of lateral ocellus. Type specimens and other materials were deposited in the Parasitic Hymenoptera Collection of the Zhejiang University, Hangzhou, China (ZJUH) and Institute of Zoology, Chinese Academy of Sciences, Beijing, China (IZCAS), respectively.

Descriptions and measurements were made under a stereomicroscope (Zeiss Stemi SV 6, www.zeiss.com ). All figures were made by a QImaging 3.3 RTVcamera ( www.qimaging.com ) attached to a stereomi croscope (Leica, ww.leica- microsystems.com) and Auto-Montage Proversion 5.0 software (Syncroscopy, www.syncroscopy.com ).

### Nomenclature

This publication and the nomenclature it contains have been registered in ZooBank. The LSID number is: urn:lsid:zoobank.org :pub:D50AE7AE-BB26-42C7-9D9D-25887EE8684F

### Taxonomy

*Spasskia brevicarinata* Yan et Chen, sp. n. ( Figures 1-11 )

**Figures 1–11.**
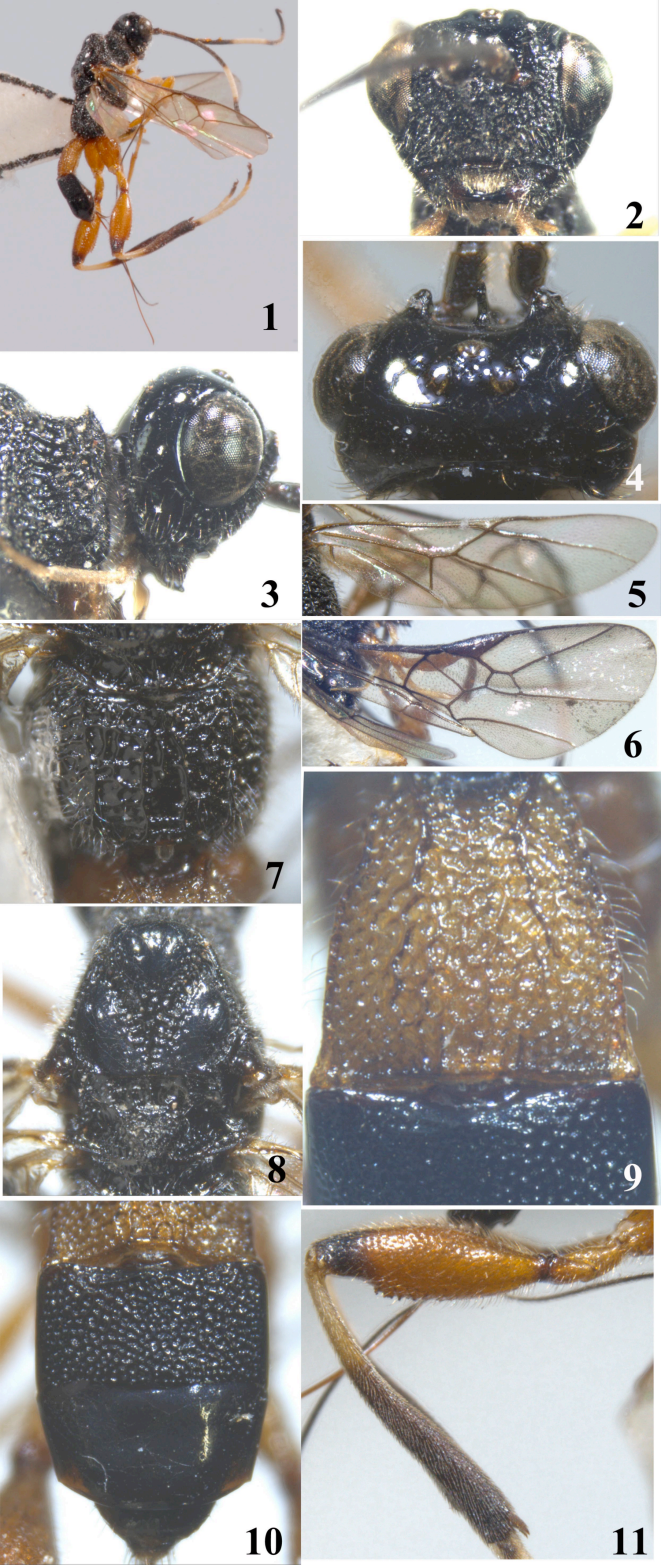
*Spasskia brevicarinata* sp. n.1. habitus, lateral aspect; 2. head, frontal aspect; 3. head, lateral aspect; 4. head, dorsal aspect; 5. hind wing; 6. fore wing; 7. propodeum, dorsal aspect; 8. mesosoma, dorsal aspect; 9. first abdominal tergite, dorsal as pect; 10. second and third metasomal tergites, dorsal aspect; 11. hind femur and tibia, lateral aspect. High quality figures are available online.

### Material examined.

Holotype: $, China, Guizhou Prov., Huishui, 2.VI.1986, Chu Jim-ing, No. 861708 (ZJUH). Paratypes: 1$, Guizhou Prov., Huishui, 2.VI.1986, Chu Jim-ing, No. 861712 (ZJUH); 1$, China, Yunnan Prov., Xinping, 1981, Jiang Yongqing, No. 815746 (ZJUH); 1$, China, Yunnan Prov., Xishuangbanna Damenglong, 650 m, 25.IV.1958, Pu Fuji, No. 1689989 IOZ(E) (IZCAS).

### Description.

Female, body length (excluding ovipositor sheath) 8.2 mm, length of extended part of ovipositor 5.5 mm, fore wing length 7.3 mm. Head. Antennal segments 35, length of third segment 1.2 times fourth segment; length of first, second, and third segments 1.7, 0.8, and 3.0 times their widths, respectively. Maxillary palp 6-segmented. Labial palp 4-segmented. Head in front view 0.8 times as long as wide. Length of eye 1.5 times temple in dorsal view. Length of malar space 0.7 times basal width of mandible, 0.4 times maximum width of eye. POL:OD:OOL = 8:15:11.

Occipital carina distinct and complete, fused with hypostomal carina below lower base of mandible. Vertex smooth and shiny. Frons crest-shaped, elevated, and smooth latero-dorsally, rugose lateroventrally, medially striated with a strongly protruding lamella. Face coarsely rugose. Clypeus rugose, weakly convex. Temple smooth and shiny, rugose near mandible. Mandibles basally striated. Mesosoma. Length almost twice as long as its height. Mesoscutum slightly longer than wide. Precoxal sulcus wide, narrowly punctate-areolate, and deep posteriorly. Pronotum largely coarsely crenulate to rugose-reticulate. Pronope present. Notauli deep and coarsely crenulate. Mesoscutum smooth. Scutellum densely punctate. Scutellar sulcus with one carina and several lateral crenulae. Metanotum with a complete median carina. Propodeum coarsely rugose-reticulate. Wings. Fore wing 3.5 times as long as wide. 1-M curved slightly. Pterostigma 3.4 times as long as wide. r:3-SR:SR1 = 11:8:61. 2-SR:3-SR:r-m = 7:4:5. 1-M:m-cu = 33:15. SR1 straight. Hind wing, marginal cell widened apically. cua inclivous. Legs. Length of femur, tibia, and basitarsus of hind leg 2.8, 7.6, and 5.0 times their widths, respectively. Hind femur robust and with a wide keel ventrally. Length of outer and inner hind tibial spurs 0.28 and 0.25 times basitarsus, respectively. Metasoma. First tergite widened posteriorly, rugose-reticulate, dorsal carinae distinct in basal one-third; length of first tergite 1.2 times its apical width. Second tergite coarsely punctate, about twice as wide as long. Third tergite with scattered punctures basolaterally, but smooth medially and posteriorly. Ovipositor sheath 1.5 times as long as metasoma, 1.3 times as long as hind tibia, 1.2 times as long as mesosoma, and 1.4 times as long as fore wing. Colour: Black. Palp yellow. Antenna dark brown (basal fourth reddish brown), but 11th-15th flagellomeres whitish yellow. Tegulae dark brown. Fore and middle legs yellow; hind coxa, trochanters, and femur light reddish brown; basal four segments of hind tarsus and basal one-third of hind tibia whitish yellow. First tergite yellow. Pterostigma and most veins dark brown, wing membrane faintly fumose.

### Male.

Unknown.

### Variation.

Body length 9.0–9.8 mm. Fore wing length 7.8–7.9 mm. Fore wing 3.1–3.5 times as long as maximum width. r:3-SR:SR1 = 9:10:51. 2-SR:3-SR:r-m = 12:10:10. Dorsal carinae of first tergite distinct in basal 0.2.

### Diagnosis.

This new species is similar to *S. indica* , but differs in having the first tergite with two short dorsal carinae at base (the latter with two complete dorsal carinae); third tergite with scattered punctures basolaterally, and smooth medially and posteriorly (the latter with distinctly fine punctures), and occipital carina connected near apex of hypostomal carina below level of mandible base (the latter connected near apex of hypostomal carina above level of mandible base).

### Distribution.

China (Guizhou, Yunnan).

### Etymology.

Name derived from “ *brevi* -” (Latin for “short”) and “ *carinata* ” (Latin for “carina”), meaning the dorsal carinae of the first tergite is short.

*Spasskia indica* Singh, Belokobylskij et Chauhan, 2005 ( Figures 12–20 )

**Figures 12–20.**
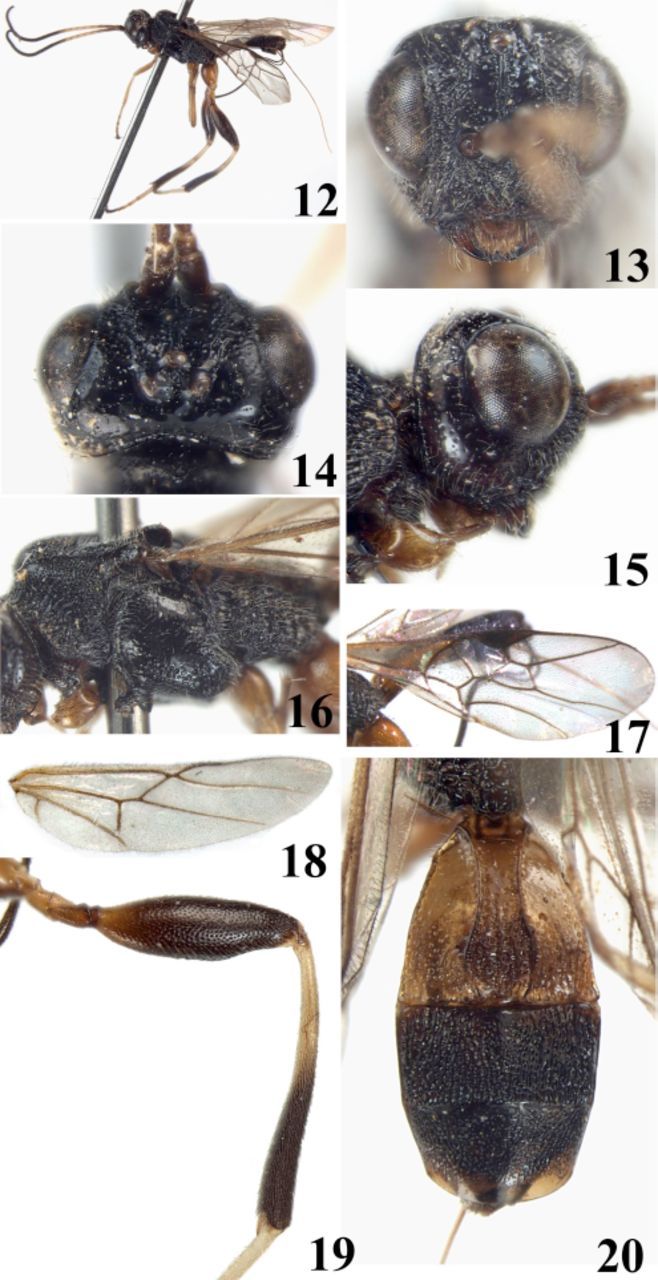
*Spasskia indica* Singh, Belokobylskij et Chauhan. 12. habitus, lateral aspect; 13. head, frontal aspect; 14. head, dorsal aspect; 15. head, lateral aspect; 16. mesosoma, lateral aspect; 17. fore wing; 18. hind wing; 19. hind femur and tibia, lateral aspect; 20. metasoma, dorsal aspect. High quality figures are available online.

### Material examined.

1♀, China, Yunnan Prov., Xishuangbanna Meng’a, 1050–1080 m, 16.X.1958, Chen Zhizi, No. 1689929 IOZ(E) (IZCAS).

### Diagnosis.

Frons crest-shaped elevated, rugose-punctate laterally, medially with a strongly protruding lamella. Occipital carina connected near apex of hypostomal carina above level of mandible base. Vertex almost smooth and shinny. Temple punctate. Malar space about 0.35 times longitudinal diameter of eye, almost equal to basal width of mandible. Precoxal sulcus short, punctate anteriorly, punctate-areolate, and deep posteriorly. Mesoscutum smooth for the most part.

### Distribution.

China (Yunnan), new record; India.

### Key to species of the genus *Spasskia*

1. Vertex and temple almost smooth; mesoscutum smooth for the most part………2
  - Vertex and temple entirely coarsely rugose-areolate; mesoscutum rugose-punctate for the most part……………………………..3
2. First tergite with two complete dorsal carinae up to the middle of tergite, the middle part between the carinae with deep irregular pits at the posterior two-thirds; lateral parts almost smooth with scattered punctures; third tergite with distinctly fine punctures; Occipital carina connected near apex of hypostomal carina above level of mandible base. China (Yunnan), India……………………………….….. **S. indica** Singh, Belokobylskij et Chauhan
First tergite rugose-reticulate, dorsal carinae distinct in basal one-third; third tergite with scattered punctures basolaterally, but smooth medially and posteriorly; Occipital carina connected near apex of hypostomal carina below level of mandible base. China (Guizhou, Yunnan)…... *S. brevicarinata* sp. n.
3. First metasomal tergite wide, roundly widened towards apex, its length slightly less than its apical width; third tergite distinctly narrowed posteriorly, coarsely sculptured, covered the following tergites; hind femur
with a fine keel ventrally; mesosoma 1.7 times as long as its height; palpi yellowish brown. Russian Far East, South Korea……………….……………….. **S. sigalphoides** Belokobylskij -First metasomal tergite narrow, almost linearly widened toward apex, its length 1.2 times as long as its apical width; third tergite weakly narrowed posteriorly, finely sculptured, not covering the following tergites; hind femur with a wide keel ventrally; mesosoma 2.2 times as long as its height; palpi dark reddish brown in basal half and yellowish brown in apical half. Russian Far East………………. *………………….S. anastasiae* Belokobylskij

## Abbreviations

POL: postocellar line
OOL: ocular-ocellar line
OD: maximum diameter of lateral ocellus
